# Estimating COVID-19 Vaccine Effectiveness for Skilled Nursing Facility Healthcare Personnel, California, USA

**DOI:** 10.3201/eid2808.220650

**Published:** 2022-08

**Authors:** Monise Magro, Andrea Parriott, Tisha Mitsunaga, Erin Epson

**Affiliations:** Author affiliation: California Department of Public Health, Richmond, California, USA

**Keywords:** COVID-19, coronavirus disease, severe acute respiratory syndrome coronavirus 2, SARS-CoV-2, coronavirus, viruses, respiratory infections, vaccine effectiveness, vaccine efficacy, vaccine-preventable diseases, skilled nursing facility, healthcare personnel, nursing home, healthcare personnel, healthcare worker, matched case‒control study, conditional logistic regression, zoonoses, United States

## Abstract

We estimated real-world vaccine effectiveness among skilled nursing facility healthcare personnel who were regularly tested for SARS-CoV-2 infection in California, USA, during January‒March 2021. Vaccine effectiveness for fully vaccinated healthcare personnel was 73.3% (95% CI 57.5%–83.3%). We observed high real-world vaccine effectiveness in this population.

The COVID-19 pandemic has severely affected skilled nursing facility (SNF) residents and healthcare personnel (HCP) (*1**,**2*). HCP are at high risk for SARS-CoV-2 exposure during patient care (*3**,**4*), and were among the earliest groups prioritized for COVID-19 vaccination starting in mid-December 2020 (*5*). Through June 2021, all SNF HCP in California, regardless of vaccination status or symptoms, were required to undergo at least weekly screening testing for SARS-CoV-2 infection (*6*). This system provided us with ideal conditions to assess vaccination effectiveness.

We estimated real-world effectiveness of COVID-19 vaccination against PCR-confirmed SARS-CoV-2 infections in SNF HCP in California by using a matched case‒control study. We identified SNF HCP COVID-19 case-patients and controls from the statewide communicable disease reporting system (Appendix). We selected persons 18–54 years of age (Appendix) with specimen collection dates during January‒March 2021. We obtained COVID-19 vaccination status from the California Immunization Registry (Appendix).

We defined partial vaccination as >1 vaccine dose received before specimen collection with a second dose (if received, for a 2-dose series vaccine) <14 days before collection, and full vaccination as the second dose (or 1 dose in a single-dose series) received >14 days before specimen collection. We matched case-patients to controls on specimen collection date and SNF county by using simple random sampling (without replacement) and a 1:1 ratio. We applied conditional logistic regression to estimate vaccine effectiveness for partial and full vaccination (compared with no vaccination).

Because of the density-based selection of the control series, in which controls are time-matched to case-patients, drawing from a risk set of persons who are at risk for becoming case-patients at the time the case is detected, the odds ratio approximates the incidence rate ratio without reliance on the rare disease assumption (*7*). We examined age, sex, and California Healthy Places Index (HPI) composite health score (*8*) by using HCP residential address and race and ethnicity (Appendix) as potential confounders.

We performed the analysis before and after excluding case-patients and controls who had previously confirmed positive test results within 90-day and 180-day windows (Table). We performed analyses by using SAS version 9.4 (https://www.sas.com). This study received an exempt determination from the California Committee for the Protection of Human Subjects.

**Table:** Estimated COVID-19 vaccine effectiveness among skilled nursing facility healthcare personnel, California, USA, January‒March 2021*

Models	Vaccination status†	No.		Vaccine effectiveness (95% CI), %
		Case-patients	Controls	
No removal of previous positive results (4,238 case‒control participants; 2,119 matched pairs)	Partial	465	629	37.5 (27.7–46.0)
	Full	36	94	71.7 (55.9–81.8)
Removal of previous positive results within 90 d (3,742 case‒control participants; 1,871 matched pairs)	Partial	430	567	35.6 (24.8–44.8)
	Full	32	89	73.3 (57.5–83.3)
Removal of previous positive results within 180 d (3,424 case‒control participants; 1,712 matched pairs)	Partial	394	524	36.3 (25.1–45.8)
	Full	25	70	72.7 (54.3–83.7)

*Unadjusted analysis results are presented. Adjustment for sex, age, and Healthy Places Index scores did not substantially alter these estimates. †Partial vaccination: >1 dose before specimen collection date but final dose <14 d before specimen collection date; full vaccination: final dose >14 d before specimen collection date.

Of the 4,238 study participants, 28.9% (1,224) were partially or fully vaccinated; 71.1% (3,014) were classified as unvaccinated, including 47.8% (2,025) who did not have a California Immunization Registry COVID-19 vaccination record and 23.3% (989) who were vaccinated on or after specimen collection date. A higher proportion of controls than case-patients were partially or fully vaccinated (Table). Among the fully vaccinated, 91.5% received Pfizer-BioNTech vaccine (https://www.pfizer.com) and 8.5% received Moderna vaccine (https://www.modernatx.com). Among the partially vaccinated, 54% received Pfizer-BioNTech vaccine, 45% received Moderna vaccine, and <1% received a combination of 2 different vaccines (e.g., Pfizer-BioNTech and Moderna). All Johnson & Johnson/Janssen vaccine (https://www.janssencovid19vaccine.com) recipients, representing 1.7% of participants matched to a vaccination record, were classified as unvaccinated because the vaccination date was after the specimen collection date.

Vaccine effectiveness was 73.3% (95% CI 57.5%–83.3%) for full vaccination (Table). We observed no substantial change (<10%) in vaccine effectiveness estimates produced by the models with or without removal of previous positive test results (Table). We assumed the model excluding previous positive test results within 90 days was the most appropriate because this model excludes persons with potential residual viral shedding and agrees with the national COVID-19 disease (new) case definition (*9*) that excludes persons who had previous positive test results within 90 days. Adjustment for age, sex, and HPI score did not change vaccine effectiveness estimates by >10%, and inclusion of race/ethnicity did not alter the full vaccination estimate by >10% (Appendix Tables 2–5).

A major strength of our study is that SNF HCP were tested regularly irrespective of symptoms or known exposure, enabling us to capture their infection status and estimate vaccine effectiveness for prevention of COVID-19, including asymptomatic infection. The unchanged vaccine effectiveness estimate after adjustment for HPI score reflects that COVID-19 vaccination efforts for SNF HCP engaged persons regardless of their residential community. One limitation is that the study period was before the Delta or Omicron virus variants became dominant. Because serial testing of vaccinated SNF HCP in California stopped during July 2021, the study period could not be expanded to examine effectiveness against later variants or changes in vaccine effectiveness over time since vaccination. In addition, a higher proportion of case-patients and controls were classified as partially vaccinated, rather than fully vaccinated, during the study period, and we did not have sufficient follow-up time to assess waning of vaccine effectiveness. Some residents could have been misclassified as HCP, but the age selection criteria limiting age group helped minimize this factor. Finally, misclassification of vaccination status is possible, but most likely is nondifferential, which we would expect to bias the odds ratio toward the null.

In conclusion, we observed high real-world effectiveness of COVID-19 vaccination in SNF HCP in California. Our methods can guide future studies evaluating vaccine effectiveness.

Appendix Additional information on estimating COVID-19 vaccine effectiveness for skilled nursing facility healthcare personnel, California, USA.
