# Supplementary material for: Estimating COVID-19 Vaccine Effectiveness for Skilled Nursing Facility Healthcare Personnel, California, USA
Source: Emerg Infect Dis. 2022 Aug;28(8):1734–6. doi: 10.3201/eid2808.220650 (PMC9328913; doi:10.3201/eid2808.220650)
Supplement: Appendix — Additional information on estimating COVID-19 vaccine effectiveness for skilled nursing facility healthcare personnel, California, USA. [file 22-0650-Techapp-s1.pdf]

Article DOI: https://doi.org/10.3201/eid2808.220650

Estimating COVID-19 Vaccine Effectiveness for Skilled Nursing Facility Healthcare Personnel, California, USA

Appendix

Healthcare Personnel Definition

Healthcare personnel (HCP) is a broad category that includes anyone working at a skilled nursing facility (SNF) (both paid and unpaid) who has the potential for direct or indirect exposure to residents, including but not limited to nursing, environmental services, and administrative staff.

Age Selection Criteria

We limited the analysis to include subjects 18–54 years of age to minimize the risk for misclassifying SNF residents as HCP. The age cutoff was determined based on the SNF HCP case data age distribution (before selecting the study period January–March 2021) and the upper cutoff represented the 75th percentile. Although this conservative approach excluded older HCP, it still captured most HCP.

Data Sources and Preparation

California Reportable Disease Information Exchange (CalREDIE)

We obtained cases and controls from the COVID-19 case registry (containing patient demographic, laboratory, epidemiology, and case investigation information) and COVID-19 positive and negative (PCR-electronic laboratory reporting (ELR) test results (ELR test results dataset), from data in CalREDIE, the California Department of Public Health (CDPH) electronic notifiable communicable disease reporting and surveillance system (*1*) merged with data reported from San Diego and Los Angeles Counties (which use separate disease reporting systems). Most samples were collected by nasopharyngeal or nasal swabs by staff at the skilled nursing facility and submitted to their preferred laboratory for PCR testing for detection of SARS-CoV-2.

Cases

We identified SNF HCP by using a deterministic matching algorithm developed by CDPH staff (COVID-19 congregate setting case dataset). The algorithm loops through all relevant CalREDIE incident and outbreak field sets. The algorithm first tries to link each case to a congregate setting facility by standardizing the case address and matching based on a dataset of congregate healthcare, correctional, educational, and childcare facility addresses. If there is no match to a specific facility, the algorithm looks for a congregate setting type based on drop down selection choices and open-text key word searches by using language standardized in a congregate setting cross reference. The algorithm also searches specific congregate settings and occupation fields to assign whether the case is a staff, resident, or student of a congregate setting. To obtain the most appropriate specimen collection date, we matched the HCP case data to COVID-19 positive PCR ELR test results based on the CalREDIE disease incident identification number (incident ID) associated with the laboratory report. If more than one positive PCR ELR test result was reported for the same incident ID within the study period (January–March 2021), we selected the test result with the earliest collection date. We performed a combination of manual review and fuzzy matching (i.e., edit distance) to search different CalREDIE free-text fields for a SNF name or address when the provided congregate setting dataset was able to capture cases associated with a SNF congregate setting but a SNF name and facility ID was not detected with their address algorithm. In those cases, we calculated edit distance between the CalREDIE congregate setting name field and the SNF name obtained from the CDPH Licensing and Certification healthcare facility list [publicly available at the California Health and Human Services Open Data Portal (*2*)] to assist in the identification of potential SNF names, which we then manually reviewed records with a compged (*3*) score <2000, spedis (*4*) score <30 or a Jaro-Winkler distance (*5*) >0.79. These cut off values used in these metrics were determined from manual inspection of the data and were considered a reasonable threshold that could contain a SNF name match. We excluded SNF co-located with a residential care facility to avoid selecting HCP working at the residential care part of the facility who were not required to receive regular screening tests. Because San Diego County uses its own disease reporting system and does not report into CalREDIE, it was underrepresented in the case data and therefore removed from the analysis. There were 2,159 eligible SNF HCP cases.

Controls

We obtained controls from persons who had COVID-19 negative PCR ELR test results using an address standardization algorithm adapted from the algorithm used to produce the COVID-19 case congregate setting dataset since the occupation field was rarely completed in the ELR test results dataset. We standardized the ELR residential address and facility address fields, as well as the SNF addresses of 1,044 included SNF licensed by the CDPH Licensing and Certification Program. We followed the same selection/exclusion criteria applied for the cases by maintaining only those records with 18–54 years of age working in SNF located in the same counties present in the eligible case-dataset, and excluding SNF associated with a residential care facility. Duplicate records, incomplete and out-of-state addresses, persons experiencing homelessness, or those missing a residential or facility address were excluded from the analysis. We matched the ELR negative test result facility address with the SNF address keeping only records with a residential address that was different from the facility address. To ensure the difference was not due to an address typo or misspelling, we applied fuzzy matching (i.e., edit distance) to quantify how dissimilar two strings were from each other (residential address compared with facility address). The cut off values used for the edit distance metrics in the control group were determined from manual inspection of the data and were considered a reasonable threshold that could contain an address match. We flagged records that had within the residential address an identifier that indicated a residential address (e.g., apartment, unit, space numbers) or a potential business/commercial address (i.e., suite number). Using a conservative approach, we excluded records if

• a business identifier was present; or

• compged score was ≤400 and spedis score ≤28; or

• Jaro-Winkler distance was >0.85 and records had the same street name (or number) and zip code, and a residential identifier was not present; or

• the street name and number were the same but not necessarily the zip code (to further exclude records that might have misspellings in zip code but otherwise had the same address).

To ensure exclusion of congregate setting residents and other healthcare facility patients, we also applied fuzzy matching to quantify how dissimilar the residential addresses were compared with addresses from licensed healthcare facilities, facilities regulated by the California

Department of Social Services (e.g., adult residential care facilities), and correctional facilities (e.g., county jails, prisons, detention centers, and pre-trial facilities). We flagged records with compged score ≤500 or spedis ≤21 and persons residing in the same residential address, which could be indicative of a congregate setting and not a residential address for further review and exclusion. We excluded records if

• the street number and name were the same; or

• the street number, zip code, and the street number of a street name (i.e., this only applied to street names that had numbers as part of their name such as ‘35TH ST’) were the same; or

• the street number was the same, the facility address contained a residential identifier (e.g., unit number) and the residential address was flagged to have a residential identifier.

We created a unique individual identifier by using first and last names and date of birth since we did not have a unique person identifier in the available dataset and the incident ID in which the laboratory report was associated with was not commonly the same across multiple tests for the same individual. We used the created unique person identifier to further exclude records if more than 2 persons resided in the same address and no residential identifier present (e.g., apartment number) for a least one of the records to increase the probability that selected controls were HCP rather than congregate setting residents. There were 344,930 eligible SNF HCP controls. Eligible controls were not person-specific, but test-specific, so the same individual could be in the control group more than once if the specimen collection date was unique.

Identification and Flagging of Previous Positive Test Results in Eligible Cases and Controls

We identified prior positives in both eligible cases and controls by using the positive PCR ELR tests dataset selecting the specimen collection date from July, 2020 through March, 2021. We did an exact match on first and last names (variable names were standardized: uppercased and spaces and characters removed), and DOB. We flagged records with a positive test within 90 days (which accounted for 10.3% of the eligible cases and 2.5% of the eligible controls) and 180 days (13.9% of the eligible cases and 8.3% of the eligible controls) for later exclusion in subsequent conditional logistic regression analyses.

Case–Control Selection and County Representation

We matched cases and controls based on specimen collection date and county of the SNF (which could be different than the county of HCP residence). Of 2,159 eligible cases, 2,119 (98.1%) matched to a control subject and were included in the analysis for a total of 4,238 case-control subjects. A total of 39 (69.6%) of 56 California counties with a SNF present and 772 (73.9%) of 1,044 licensed SNF (excluding those associated with a residential care facility and from San Diego County) were included in the case-control matched pair dataset. Excluded counties comprised San Diego due to incomplete data in the cases and some northern counties (typically small rural counties with small numbers of SNF) with no matching controls due to low test counts. A total of 10 counties accounted for 84.8% (n = 3592) of the case-control subjects with Los Angeles County accounting for 43.3% (n = 1834) of the case-control subjects (Appendix Table 1).

COVID-19 Vaccine Data

ELR positive and negative test results were linked to the California Immunization Registry (CAIR) by CDPH staff applying similar methodology used for the identification of California COVID-19 post-vaccination cases and were matched based on data received and processed through early August 2021. A probabilistic match approach was completed with R software (https://www.r-project.org/) using the RecordLinkage package; records were matched based on an exact match on zip code of residence and date of birth, and a fuzzy match on first and last name (variable names were standardized: uppercased and spaces and special characters were removed). We manually reviewed all one-to-many records and any records with a weight ≤0.9525 to verify whether they were an actual match (retaining the match with the highest weight if two vaccine records were matched to an individual) or if a data entry error caused the individual vaccine record to be split into two vaccine records (e.g., first dose in one record and the second dose in a different record), requiring them to be merged into one vaccine record. Validating the matched records involved examining additional variables (i.e., sex and residential address) available in CAIR against the case-control dataset. CDPH decided on 0.9525 as the "match" probability threshold for post-vaccination case identification. Thus, when a laboratory record matched to a CAIR record with a probability of 0.9525 or higher, this record would be judged a "match" and would count as a post-vaccination case. The high threshold of 0.9525 was chosen after extensive manual review to minimize the risk for records of different persons being

matched and resulting in inflated post-vaccination case counts. Over 98.7% of the case-control subjects that were matched to a vaccine record had a weight >0.9525, 84.1% a weight of 1 (exact match) and only 1.3% a weight ≤0.9525 (range: 0.9–0.95). After data cleaning, records were linked to the case-control matched dataset by using the CalREDIE disease incident ID that the laboratory report is associated with. The Appendix Figure summarizes the main data processes and methods used for the identification of SNF HCP for inclusion in this study.

California Healthy Places Index (HPI) Data

We evaluated a composite health equity vulnerability measure (California HPI score) (*6, 7*) as a potential confounder in our regression model (Appendix Table 2). Healthy Places Index data are publicly available at the census tract level, although not all census tracts are given an HPI score (*8*). To obtain census tracts for each case-control subject, we standardized residential addresses and geocoded them by using the Census geocoder (*9*). We used ArcGIS (https://www.esri.com/en-us/arcgis/products/arcgis-desktop/overview) by using ArcMap version 10.7.1 to geocode any addresses not matched by using the Census geocoder. Only 102 (2.4%) subjects' addresses could not be geocoded and did not have a census tract assigned. We assigned 4,136 (97.6%) case-control subjects to a census tract and matched 3,388 (79.9%) case-control subjects to census tracts with an HPI score. Of those not matched by census tract, we were able to match 817 (19.3%) subjects to an HPI score by zip code. For 33 (0.8%) subjects that did not match previously (e.g., had an incomplete address to be geocoded, were missing a zip code, or did not have an HPI score calculated), we calculated an HPI weighted average by county of residence weighted by population at the county by using the population variable available at the HPI in the census tract file.

Race and Ethnicity

We assigned the variable race/ethnicity as Hispanic or Latino (regardless of the race reported) to subjects that reported 'Hispanic or Latino' for ethnicity; otherwise, we assigned race/ethnicity according to the race reported. We combined Black or African American, American Indian or Alaska Native, Native Hawaiian or Other Pacific Islander, and Multi-race into one race/ethnicity category due to small sample size concerns. Although the races from the combined category could experience different COVID-19 disease risk, the race/ethnicity categorization was used as a covariate and not as the primary exposure of interest, and not intended for interpretation of their odds ratio. We set race/ethnicity to missing when not

available, not informative (i.e., “Unknown”), or when reported as “Other.” We set “Other” race/ethnicity category to missing as a conversative approach due to concerns about data quality (i.e., mistakenly reporting “Other” instead of “Unknown”). Data for race/ethnicity was missing for a high proportion of the subjects (22% in cases and 48% in controls). Thus, when accounting for race/ethnicity in the model, 59.1% (based on dataset without removal of prior positive tests within 90- and 180-day windows) of the study subjects were excluded as the entire matched case-control pair is removed in a conditional logistic regression when a covariate is missing for one of the matched pair subjects (Appendix Table 3). After adjustment of race/ethnicity, the VE estimate for full vaccination slightly increased in all models, ranging from 76.1 to 82.8% (increase ranged from 5% to 13.9% compared with the VE for the respective unadjusted models) and partial vaccination decreased, ranging from 20.3% to 24.9% (decrease ranged 31.4% to 45.9% decrease compared with the VE for the respective unadjusted models) depending on the model (Appendix Table 3). The observed VE estimate variability is likely due to the high proportion of observations removed from the analysis, which considerably reduced sample size and widened VE estimate confidence intervals, making adjustment of this covariate by using the available data not ideal. Thus, we examined the use of demographic race/ethnicity data from the American Community Survey (ACS) (5-year estimate, 2015–2019 at the census tract level) (https://www.census.gov/programs-surveys/acs). We assigned race/ethnicity as ‘Hispanic or Latino’ (regardless of race) when ethnicity indicated ‘Hispanic or Latino’, otherwise we assigned the race reported. We obtained the race/ethnicity with the highest proportion for each census tract. After merging these data to the study subjects’ census tracts, only Hispanic or Latino, White, Asian, and Black or African American were within the race/ethnicity categories observed. We adjusted the model by race/ethnicity by using ACS data (data not shown) and the VE estimates decreased by 3.5% for partial vaccination and 0.4% for full vaccination compared with the unadjusted model. We compared race/ethnicity generated from ACS data with the race/ethnicity categories generated from the case-control subject data (when available) and observed low concordance (<50%). Considering the diverse race/ethnicity population in California and the low concordance observed, we determined that it would not be suitable to apply the adjustment by using ACS data in the final model.

References

1. California Department of Public Health. California Reportable Disease Information Exchange. [cited 2022 Jan 27] https://www.cdph.ca.gov/Programs/CID/DCDC/Pages/CalREDIE.aspx

2. California Health and Human Services Agency Open Data. Licensed Healthcare Facility Listing [cited 2022 Jan 11] https://data.chhs.ca.gov/dataset/licensed-healthcare-facility-listing

3. SAS Help Center. Functions and call routines. COMPGED function [cited 2022 Jan 11] https://documentation.sas.com/doc/en/vdmmlcdc/8.1/lefunctionsref/p1r4l9jwgatggtn1ko81fyjys4s7.htm

4. SAS Help Center. Functions and call routines. SPEDros information service function [cited 2022 Jan 11] https://documentation.sas.com/doc/en/vdmmlcdc/8.1/lefunctionsref/p0vmuxh8ljfn7on164nsgvmdrc5d.htm

5. Cohen WW, Ravikumar P, Fienberg SE. A comparison of string distance metrics for name-matching tasks. IIWeb; 2003: Citeseer; 2003. p. 73–8 [cited 2022 May 26. https://www.cs.cmu.edu/~wcohen/postscript/ijcai-ws-2003.pdf

6. Public Health Alliance of Southern California. The California Healthy Places Index (HPI). About. [cited 2022 Jan 11] https://healthyplacesindex.org/about

7. Maizlish N, Delaney T, Dowling H, Chapman DA, Sabo R, Woolf S, et al. California healthy places index: frames matter. Public Health Rep. 2019;134:354–62. PubMed https://doi.org/10.1177/0033354919849882

8. Public Health Alliance of Southern California. The California Healthy Places Index (HPI). Data and Reports. [cited 2022 Jan 11] https://healthyplacesindex.org/data-reports

9. United States Census Bureau. Welcome to Geocoder [cited 2022 Jan 12]. https://geocoding.geo.census.gov

Appendix Table 1. Top 10 California counties and skilled nursing facilities represented in case-control matched pair dataset

Counties		Case–control subjects		Skilled nursing facility (SNF)			
Top 10	Name	No.	%	No. in study	% in study	No. of total available*	% represented†
1	Los Angeles	1,834	43.3	306	39.6	371	82.5
2	Alameda	284	6.7	55	7.1	67	82.1
3	Orange	268	6.3	49	6.3	64	76.6
4	Contra Costa	252	5.9	29	3.8	29	100
5	Sacramento	230	5.4	34	4.4	35	97.1
6	Santa Clara	220	5.2	38	4.9	45	84.4
7	Sonoma	196	4.6	13	1.7	18	72.2
8	San Bernardino	108	2.5	31	4.0	51	60.8
9	Fresno	102	2.4	24	3.1	28	85.7
10	San Joaquin	98	2.3	16	2.1	25	64
	Other counties	646	15.2	177	22.9	285	62.1
	Total	4,238	100.0	772	100.0	1,018	

*Total SNF (available) located in each county excluding those associated with a residential care facility.
†Calculated as number of SNF in the study in each county divided by SNF available (located) in each county, per 100.

Appendix Table 2. Estimated COVID-19 vaccine effectiveness among California skilled nursing facility healthcare personnel, adjusted by Healthy Places Index score based on reported data, January–March 2021

Models	Variables	No.		VE* and 95% CI† (%)	OR and 95% Wald CI	p value
		Case-patients	Controls			
No removal of prior positives (4,238 case-control subjects; 2,119 matched pairs)	Vaccination status					
	Partial	465	629	37.4 (27.5–46.0)	0.63 (0.54–0.73)	<0.0001
	Full	36	94	71.6 (55.7–81.7)	0.28 (0.18–0.44)	<0.0001
	HPI score‡	2,119	2,119		0.85 (0.73–0.99)	0.0403
Removal of prior positives within 90 d (3,742 case-controls subjects; 1,871 matched pairs)	Vaccination status					
	Partial	430	567	35.5 (24.7–44.7)	0.65 (0.55–0.75)	<0.0001
	Full	32	89	73.2 (57.3–83.2)	0.27 (0.17–0.43)	<0.0001
	HPI score	1,871	1,871		0.9 (0.76–1.05)	0.1801
Removal of prior positives within 180 d (3,424 case-controls subjects; 1,712 matched pairs)	Vaccination status					
	Partial	394	524	36.2 (25–45.7)	0.64 (0.54–0.75)	<0.0001
	Full	25	70	72.5 (54.0–83.6)	0.28 (0.16–0.46)	<0.0001
	HPI score	1,712	1,712		0.89 (0.76–1.06)	0.1914

*Vaccine effectiveness.
†Confidence intervals.
‡Healthy Places Index score.

Appendix Table 3. Estimated COVID-19 vaccine effectiveness among California skilled nursing facility healthcare personnel, adjusted by race and ethnicity based on reported data, January–March 2021

Models	Variables	No.		VE* and 95% CI† (%)	OR‡ and 95% Wald CI	p value
		Case-patients	Controls			
No removal of prior positives (1,732 case-control subjects; 866 matched pairs)	Vaccination status					
	Partial	217	251	20.3 (0.1–36.4)	0.80 (0.64–1.0)	0.0493
	Full	12	33	76.1 (46.7–89.3)	0.24 (0.11–0.53)	0.0005
	Race/ethnicity§					
	Hispanic or Latino	406	313		Reference	
	Asian	222	236		0.75 (0.58–0.97)	0.0266
	Combined races	115	109		0.73 (0.53–1.02)	0.0638
	White	123	208		0.43 (0.32–0.57)	<0.0001
Removal of prior positives within 90 d (1,532 case-control subjects; 766 matched pairs)	Vaccination status					
	Partial	195	228	21.3 (−0.1 to 38.2)	0.79 (0.62–1.0)	0.0513
	Full	11	31	77.0 (46.2–90.2)	0.23 (0.1–0.54)	0.0007
	Race/ethnicity					
	Hispanic or Latino	352	277		Reference	
	Asian	199	215		0.76 (0.58–0.99)	0.0431
	Combined races	102	96		0.73 (0.51–1.04)	0.0818
	White	113	178		0.47 (0.35–0.64)	<0.0001
Removal of prior positives within 180 d (1,378 case-control subjects; 689 matched pairs)	Vaccination status					
	Partial	174	208	24.9 (3.3–41.7)	0.75 (0.58–0.97)	0.0265
	Full	5	20	82.8 (48.1–94.3)	0.17 (0.06–0.52)	0.0018
	Race/ethnicity					
	Hispanic or Latino	312	244		Reference	
	Asian	181	193		0.76 (0.58–1.01)	0.0612
	Combined races	90	86		0.72 (0.49–1.05)	0.0833
	White	106	166		0.46 (0.34–0.64)	<0.0001

*Vaccine effectiveness.
†Confidence intervals.
‡Odds ratio.
§Combined races included Black or African American, American Indian or Alaska Native, Native Hawaiian or Other Pacific Islander, and Multi-race.

Appendix Table 4. Estimated COVID-19 vaccine effectiveness among California skilled nursing facility healthcare personnel, adjusted by age based on reported data, January–March 2021

Models	Variables	No.		VE* and 95% CI† (%)	OR‡ and 95% Wald CI	p value
		Case-patients	Controls			
No removal of prior positives (4,238 case-control subjects; 2,119 matched pairs)	Vaccination status					
	Partial	465	629	37.0 (27.0–45.6)	0.63 (0.54–0.73)	<0.0001
	Full	36	94	71.0 (54.8–81.4)	0.29 (0.19–0.45)	<0.0001
	Age	2,119	2,119		1.00 (0.99–1.00)	0.2206
Removal of prior positives within 90 d (3,742 case-control subjects; 1,871 matched pairs)	Vaccination status					
	Partial	430	567	35 (24.1–44.4)	0.65 (0.56–0.76)	<0.0001
	Full	32	89	72.7 (56.4–82.9)	0.27 (0.17–0.44)	<0.0001
	Age	1,871	1,871		1.00 (0.99–1.00)	0.236
Removal of prior positives within 180 d (3,424 case-control subjects; 1,712 matched pairs)	Vaccination status					
	Partial	394	524	35.4 (24.0–45.1)	0.65 (0.55–0.76)	<0.0001
	Full	25	70	71.7 (52.5–83.1)	0.28 (0.17–0.48)	<0.0001
	Age	1,712	1,712		1.00 (0.99–1.00)	0.121

*Vaccine effectiveness.
†Confidence intervals.
‡Odds ratio.

Appendix Table 5. Estimated COVID-19 vaccine effectiveness among California skilled nursing facility healthcare personnel, adjusted by sex based on reported data, January–March 2021

Models	Variables	No.		VE* and 95% CI† (%)	OR‡ and 95% Wald CI	p value
		Case-patients	Controls			
No removal of prior positives (3,422 case-control subjects; 1,711 matched pairs)	Vaccination status					
	Partial	387	512	36.0 (24.7–45.6)	0.64 (0.54–0.75)	<0.0001
	Full	36	91	70.5 (54.0–81.1)	0.30 (0.19–0.46)	<0.0001
	Sex					
	F	1,315	1,274		Reference	
	M	396	437		0.87 (0.74–1.02)	0.0822
Removal of prior positives within 90 d (3,062 case-control subjects; 1,531 matched pairs)	Vaccination status					
	Partial	361	466	33.9 (21.6–44.3)	0.66 (0.56–0.78)	<0.0001
	Full	32	86	72.2 (55.5–82.6)	0.28 (0.17–0.45)	<0.0001
	Sex					
	F	1,174	1,139		Reference	
	M	357	392		0.87 (0.74–1.03)	0.11
Removal of prior positives within 180 d (2,798 case-control subjects; 1,399 matched pairs)	Vaccination status					
	Partial	332	434	34.9 (22.2–45.5)	0.65 (0.55–0.78)	<0.0001
	Full	25	67	71.2 (51.7–82.8)	0.29 (0.17–0.48)	<0.0001
	Sex					
	F	1,074	1,035		Reference	
	M	325	364		0.85 (0.72–1.02)	0.0774

*Vaccine effectiveness.
†Confidence intervals.
‡Odds ratio.

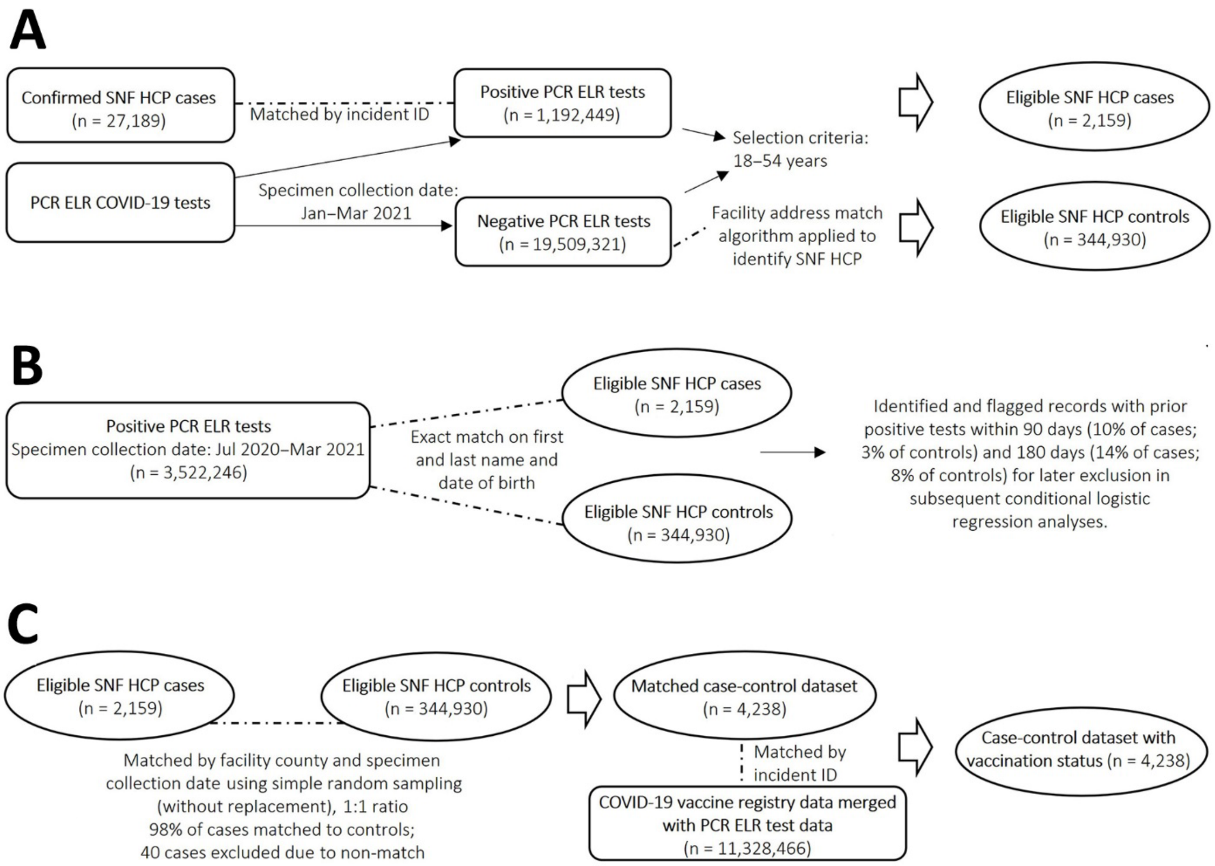

Appendix Figure. Flow diagram summarizing main data process and methods for identification of skilled nursing facility (SNF) healthcare personnel (HCP) for inclusion in COVID-19 vaccine effectiveness study in California, January–March 2021. A) Data processing and identification of eligible SNF HCP COVID-19 cases and controls; B) Identification of previous COVID-19 positive test results in eligible cases and controls; C) Case-control selection and vaccination match. COVID-19, coronavirus disease; PCR, polymerase chain reaction. ELR, electronic laboratory reporting. IncidentID, California Reportable Disease Information Exchange (CalREDIE) disease incident identification number associated with the laboratory report ID. Dashed lines indicate that a match occurred.
